# ABCG11 modulates cytokinin responses in *Arabidopsis thaliana*

**DOI:** 10.3389/fpls.2022.976267

**Published:** 2022-07-25

**Authors:** Qianying Yang, Jie Zhang, Mikiko Kojima, Yumiko Takebayashi, Takuya Uragami, Takatoshi Kiba, Hitoshi Sakakibara, Youngsook Lee

**Affiliations:** ^1^Department of Life Sciences, POSTECH, Pohang, South Korea; ^2^International Research Centre for Environmental Membrane Biology, Foshan University, Foshan, China; ^3^RIKEN Center for Sustainable Resource Science, Yokohama, Japan; ^4^Graduate School of Bioagricultural Sciences, Nagoya University, Nagoya, Japan

**Keywords:** ABC transporters, ABCG11, ABCG14, heterodimer, phytohormone, root, signaling, transport

## Abstract

The Arabidopsis ABC transporter ABCG11 transports lipidic precursors of surface coating polymers at the plasma membrane of epidermal cells. Mutants in *ABCG11* exhibit severe developmental defects, suggesting that ABCG11 might also participate in phytohormone-mediated development. Here, we report that ABCG11 is involved in cytokinin-mediated development. The roots of *abcg11* mutant seedlings failed to respond to cytokinins and accumulated more cytokinins than wild-type roots. When grown under short-day conditions, *abcg11* exhibited longer roots and shorter hypocotyls compared to wild type, similar to *abcg14*, a knockout mutant in a cytokinin transporter. Treatment with exogenous *trans*-zeatin, which inhibits primary root elongation in the wild type, enhanced *abcg11* primary root elongation. It also increased the expression of cytokinin-responsive Arabidopsis response regulator (*ARR*) genes, and the signal of the *TCS*::*GFP* reporter in *abcg11* roots compared to wild-type roots, suggesting that cytokinin signaling was enhanced in *abcg11* roots. When we treated only the roots of *abcg11* with *trans*-zeatin, their shoots showed lower *ARR* induction than the wild type. The *abcg14 abcg11* double mutant did not have additional root phenotypes compared to *abcg11*. Together, these results suggest that ABCG11 is necessary for normal cytokinin-mediated root development, likely because it contributes to cytokinin transport, either directly or indirectly.

## Introduction

The ATP-binding cassette (ABC) transporter ABCG11 is required for the transport of cutin and wax components to the extracellular matrix (Panikashvili et al., 2007). A mutant in Arabidopsis (*Arabidopsis thaliana*) *ABCG11* displays diverse developmental defects, including organ fusion, stunted growth, altered petal, and silique morphology, altered root suberin composition, and sterility (Bird et al., 2007; Panikashvili et al., 2007, 2010; McFarlane et al., 2010). ABCG11 is a half-size ABC protein that usually forms dimers to exert its transporter function (Verrier et al., 2008). Indeed, ABCG11 was shown to form homodimers and heterodimers with many other half-size ABCG proteins: ABCG5, ABCG9, ABCG12, and ABCG14 (Do et al., 2021). ABCG11 homodimers and ABCG11–ABCG12 heterodimers export lipidic molecules from epidermal cells to form the protective cuticle (Bird et al., 2007; McFarlane et al., 2010; Panikashvili et al., 2010). ABCG11–ABCG9 heterodimers regulate vascular development (Le Hir et al., 2013), while ABCG11–ABCG5 heterodimers mediate the transport of wax precursors in seedlings (Lee et al., 2020). ABCG11 also interacts with ABCG14 in Arabidopsis protoplasts, based on co-immunoprecipitation (Co-IP) assays (Le Hir et al., 2013). The multiple dimers involving ABCG11 might transport structurally different as well as similar substrates, and a broad substrate range for ABCG11 might explain the pleiotropic and severe phenotypes observed in *abcg11* knockout mutants (McFarlane et al., 2010).

Among the many binding partners of ABCG11, ABCG14 is unique in its role in the root-to-shoot translocation of the plant hormone *trans*-zeatin (tZ)-type cytokinins (Ko et al., 2014; Zhang et al., 2014). Cytokinins are a group of plant hormones critical for regulating plant growth and development (Moubayidin et al., 2010; Tokunaga et al., 2012; Yuan et al., 2016; Mellor et al., 2017; Janečková et al., 2018; Rong et al., 2018; Vatén et al., 2018; Wybouw and De Rybel, 2019). Cytokinins undergo long-distance transport from the root to the shoot *via* the xylem, or from the shoot to the root *via* the phloem, to coordinate the growth of both shoot and root (Hirose et al., 2008; Bishopp et al., 2011). The Arabidopsis *abcg14* mutant was proposed to be defective in the long-distance transport of tZ-type cytokinins due to its dwarf shoot phenotype and low tZ-type cytokinin levels in shoots but high levels in roots. In addition, xylem exudates from *abcg14* contain much less tZ-type cytokinins than the wild type (WT; Ko et al., 2014). Since ABCG14 is also a half-size ABC protein, it should form a homodimer or heterodimer to transport cytokinins, but the identity of its binding partner for the root transport of cytokinins is unknown. Here, we report multiple lines of evidence that support ABCG11 as the partner protein for ABCG14 in mediating cytokinin-dependent development, especially of the root. We show that *abcg11* mutant is characterized by an alteration of cytokinin concentrations and cytokinin-related phenotypes. We also discuss how ABCG11 might function to modulate cytokinin-dependent root growth.

## Materials and methods

### Plant materials and growth conditions

Seeds were surface sterilized with NaOCl and placed in the dark for 2 d at 4°C for stratification before being sown on half-strength Murashige and Skoog (MS) or MGRL medium (Fujiwara et al., 1992) solidified with 0.8% (w/v) phytoagar (Duchefa Biochemie, Netherlands). The medium was supplemented with 20 nM tZ when indicated. Seedlings were grown vertically in a growth chamber (22/18°C; 16 h light/8 h dark or 8 h light/16 h dark).

Two alleles of *abcg11*, SALK_119873 and SALK_131624, were obtained from the Nottingham Arabidopsis Stock Centre. SALK_119873 was reported to be defective in the secretion of cuticular lipids (Bird et al., 2007), while SALK_131624 was reported previously as *cof1-3* (*cuticular defect and organ fusion*; Ukitsu et al., 2007). The *abcg14* mutant line (SK_15918) was previously described (Ko et al., 2014).

To generate Col-0 and *abcg11* lines expressing a cytokinin signaling marker system, the *TCS*::*GFP* (*Two Component signaling Sensor*::*green fluorescent protein*) vector was introduced into Agrobacterium (*Agrobacterium tumefaciens*) strain GV3101 (Zürcher et al., 2013) for Agrobacterium-mediated floral dip transformation of Col-0 and *abcg11/+* plants. Basta was used to select transformants. Seeds were harvested from primary transformants and used for *TCS*::*GFP* fluorescence analysis. Homozygous mutant seedlings were identified based on their characteristic organ fusion phenotype.

### Quantitative real-time PCR

Total RNA was extracted from the indicated samples using RNAiso Plus (Takara, Japan) according to the manufacturer’s protocol. First-strand cDNAs were synthesized from 2 μg RNA using the Promega reverse transcriptase kit (Promega, United States). Quantitative real-time PCR was performed using TB Green^®^ Premix Ex Taq^™^ Tli RNase H Plus (TAKARA),^1^ following the manufacturer’s instructions. The primers used for RT-qPCR are shown in Supplementary Table S1.

### Cytokinin quantification

Shoots and roots of 12-d-old seedlings grown on half-strength MS plates were harvested and immediately frozen in liquid nitrogen. Cytokinin contents were quantified from the frozen samples using ultra-performance liquid chromatography–tandem mass spectrometry (AQUITY UPLC System/XEVO-TQS; Waters) with an octadecylsilyl column (AQUITY UPLC HSS T3, 1.8 μm, 2.1 mm × 100 mm; Waters). The detailed method of extraction and quantification was described before (Kojima et al., 2009).

### 3H-tZ translocation assay

Five 12-d-old seedlings per genotype were transferred to a 48-well plate, with each well containing 900 μl of half-strength MS medium and pH adjusted to pH 5.8 with MES and KOH. Only the roots were submerged in medium. After 30 min of preincubation, ^3^H-tZ or ^3^H-H_2_O was added to the wells to a final concentration of 50 nM or 0.5 μCi/ml, respectively, and the seedlings were incubated for an additional 1 h. The shoots were harvested and their radioactivity was counted with a liquid scintillation counter. The preparation of and quality checks for ^3^H-tZ were performed, as described previously (Takei et al., 2003).

## Results

### The *abcg11* knockout mutant is altered in the expression of cytokinin-response marker genes

**Figure 1:**
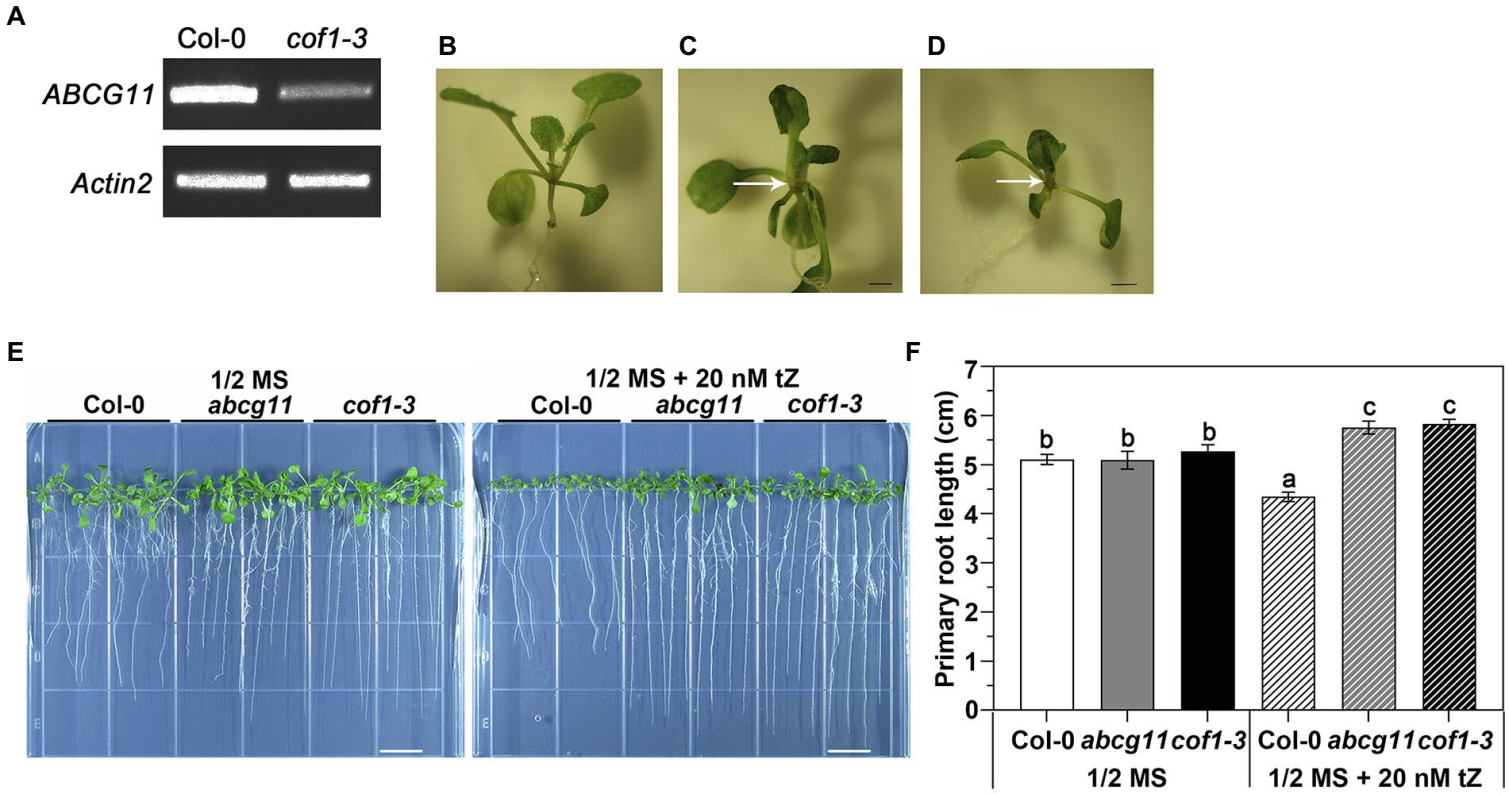
Second allele of *abcg11* T-DNA insertion line *cof1-3* (SALK_131624) exhibits an altered response to cytokinin, similar to *abcg11*. **(A)** *cof1-3* is a knockdown mutant of *ABCG11*. RT-PCR analysis of *AtABCG11*. *Actin2* (At3g18780) was amplified as an internal control. The primers are listed in Supplementary Table S3. **(B–D)** Photographs of the shoots of 12-day-old seedlings of Col-0, *abcg11*, and *cof1-3*. The arrows point the sites of organ fusion. Scale bar = 1.0 mm. **(E)** The similar responses of *cof1-3* and *abcg11* roots to 20 nM *trans*-zeatin (tZ) treatment under long-day conditions (16 h/8 h, day/night). The photographs were taken at 12 days after seed sowing. The photos are representatives of four independent experiments. Scale bars = 1 cm. **(F)** The average primary root length of the plants shown in **(E)**. The error bars represent the standard error (SE; *n* = 40). The different letters indicate the significant differences determined by one-way ANOVA (*p* < 0.05).

The *abcg11* mutant SALK_119873 was shown in previous reports to exhibit extremely severe growth retardation, multiple thin and short inflorescence stems, as well as fusion between shoots and reproductive organs (Bird et al., 2007; Luo et al., 2007; Panikashvili et al., 2007). It has been widely used in ABCG11 research. Here, this mutant is designated *abcg11*. Since the *abcg11* mutant is sterile, we used the progeny of *abcg11/+* heterozygous plants for analysis. We easily identified homozygous *abcg11* seedlings from their characteristic fusion of shoots relative to the normal appearance of WT Col-0 and *abcg11/+* seedlings (Figures 1B,C).

**Figure 2:**
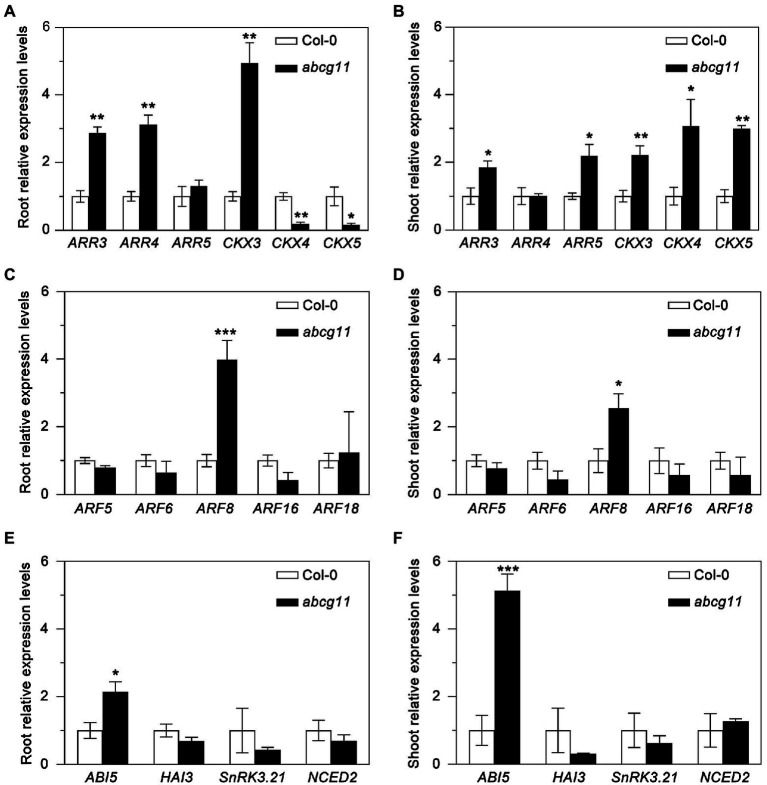
Expression of phytohormone-induced genes in Col-0 and the *abcg11* mutant. Relative expression levels of cytokinin- **(A,B)**, auxin- **(C,D)**, and abscisic acid- **(E,F)** induced genes in roots **(A,C,E)** and shoots **(B,D,E)**. Seedlings were grown vertically on half-strength MS medium for 12 d (16 h light/8 h dark). *TUBULIN8* served as an internal control; values for Col-0 were set to 1. Data are shown as means ± standard error (SE; *n* = 4). Asterisks represent significant differences by Student’s *t*-test: ^*^p < 0.05; ^**^p < 0.01; ^***^p < 0.001.

To test whether the *abcg11* mutant showed any alteration in phytohormone responses, we determined relative transcript levels for marker genes for cytokinins, auxin, and abscisic acid (ABA) in the WT and *abcg11* mutant seedlings. The expression of most cytokinin-response marker genes, such as *ARR3*, *CKX3*, *CKX4*, and *CKX5*, differed between *abcg11* and the WT in both roots and shoots (Figures 2A,B). By contrast, only one auxin-response (*ARF8*) and one ABA-induced gene (*ABI5*) were expressed to different levels in the two genotypes (Figures 2C–F), prompting us to focus on cytokinins by measuring their concentrations in roots and shoots from the WT and *abcg11*.

### Concentrations of several cytokinins are higher in *abcg11* than in the WT

**Figure 3:**
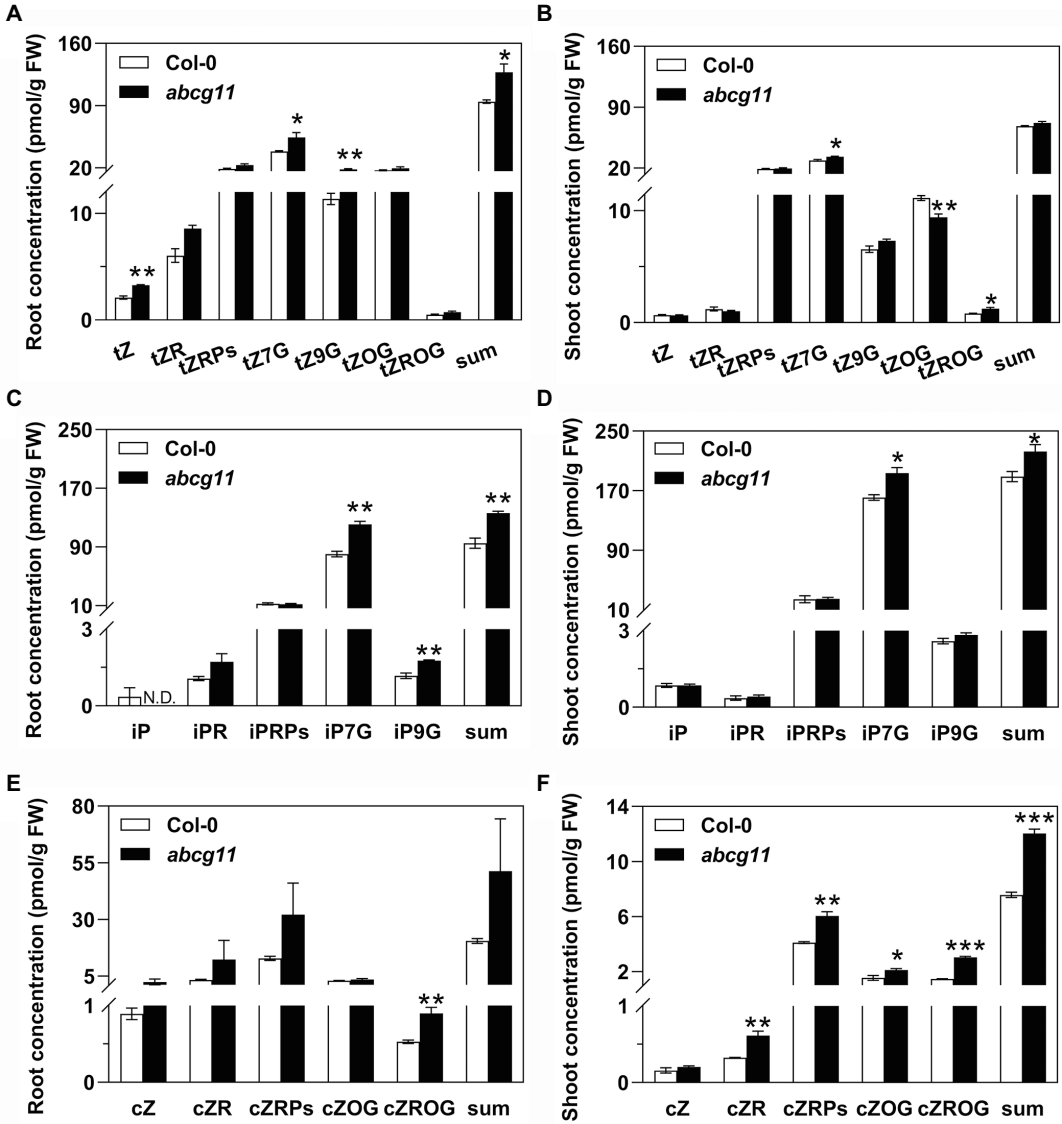
The concentration of several cytokinin types is higher in *abcg11* than in the wild type. Concentrations for the *trans*-zeatin (tZ)-type **(A,B)**, isopentyladenine (iP)-type **(C,D)**, and *cis*-zeatin (cZ)-type **(E,F)** cytokinins in roots **(A,C,E)** and shoots **(B,D,F)** of Col-0 and *abcg11*. Seedlings were grown on half-strength MS medium for 12 d under long-day conditions (16 h light/8 h dark). Data are shown as means ± SE (*n* = 4). Asterisks represent significant differences by Student’s *t*-test: ^*^p < 0.05; ^**^p < 0.01.

We measured the contents of the tZ-type cytokinins, isopentyladenine (iP)-type cytokinins, and *cis*-zeatin (cZ)-type cytokinins in roots and shoots of WT and *abcg11* mutant seedlings. In *abcg11* roots, the concentrations of the three tZ-types cytokinins tZ, tZ7G, and tZ9G were higher than in WT roots, resulting in a significant (*p* < 0.05) difference in the total amount of tZ-type cytokinins (Figure 3A; Supplementary Table S4). Concentrations for tZ7G and tZROG were similarly higher in *abcg11* shoots, while those of tZOG were lower, relative to WT shoots, resulting in a comparable total sum of tZ-type cytokinins in the two genotypes (Figure 2B; Supplementary Table S4). The concentrations of two iP-types cytokinin, iP7G and iP9G, were higher in *abcg11* relative to the WT in both roots and shoots (Figures 3C,D; Supplementary Table S4). While cZ-type cytokinins exhibited much lower levels than those of the other types, they accumulated to higher levels in *abcg11* shoots compared to WT shoots, but showed no significant differences in roots, with the exception of cZROG, whose concentrations were very low (Figures 3E,F; Supplementary Table S4). The overall changes in cytokinin concentrations in *abcg11* suggested that cytokinin metabolism and/or transport differ in the mutant.

### *abcg11* exhibits root phenotypes similar to those of *abcg14*, a cytokinin transporter knockout mutant

**Figure 4:**
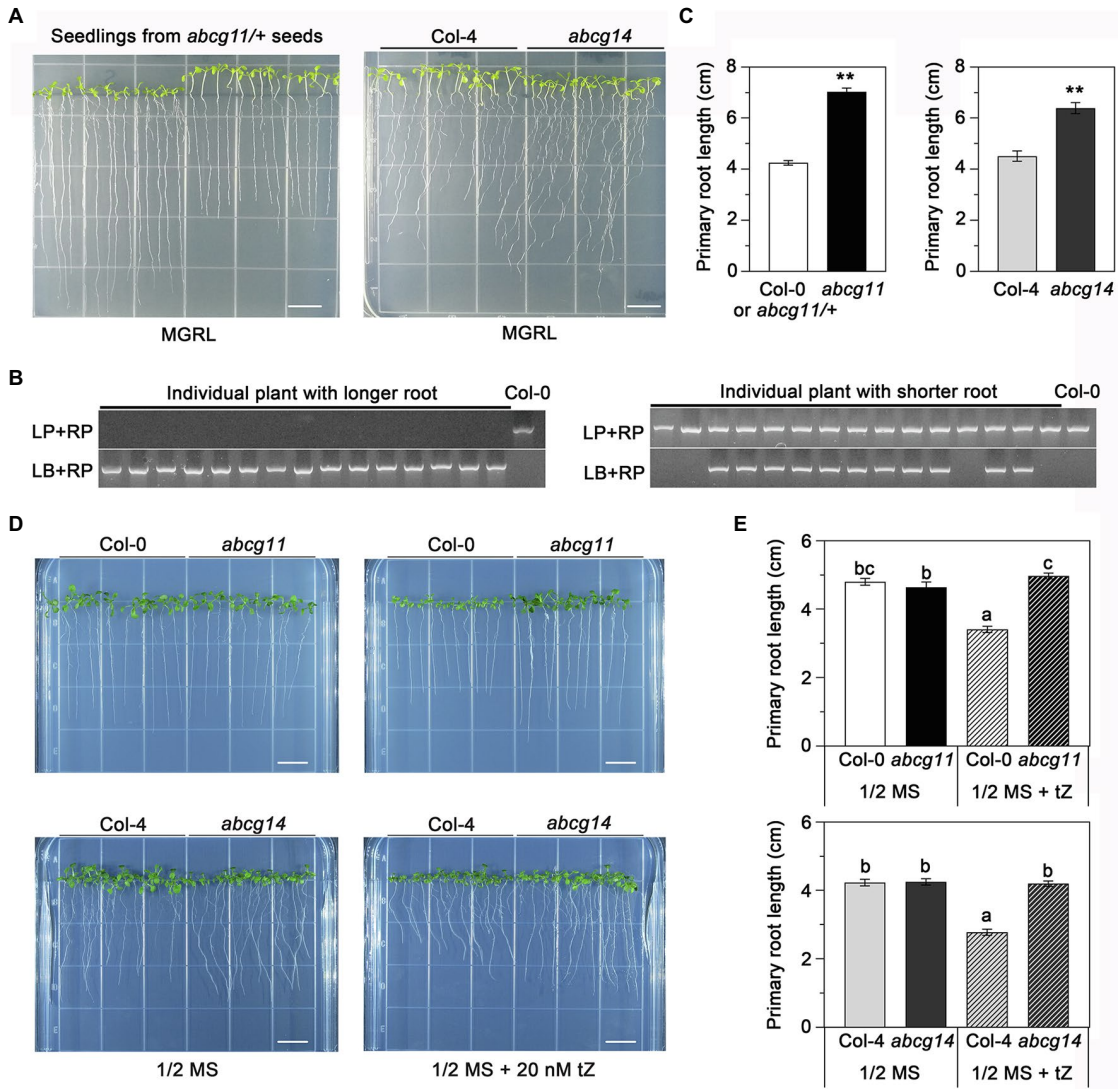
*abcg11* behaves similarly as *abcg14* in root growth on MGRL medium and in response to exogenous cytokinins. (**A**, left panel) The progeny of *abcg11/+* plants exhibit two distinct phenotypes. The seeds were sown on MGRL medium and grown vertically for 12 days under short-day conditions (8 h light/16 h dark). Note that seedlings were rearranged according to their primary root length before taking the picture. (**A**, right panel) Phenotype of *abcg14* under the same conditions. Scale bars, 1 cm. **(B)** Genotyping of the individual seedlings shown in (A), left panel. Note how the seedlings with longer roots are *abcg11* homozygous mutants. The primers for genotyping are listed in Supplementary Table S2. **(C)** Primary root length of seedlings in **(A)**. Data are shown as means ± SE (*n* = 15). Asterisks represent significant differences by Student’s *t*-test: ^**^p < 0.01. **(D)** Phenotype of seedlings grown with or without 20 nM *trans*-zeatin (tZ) for 12 days under long-day conditions (16 h light/8 h dark) on half-strength MS medium. The photographs are representative of three independent experiments. Scale bars, 1 cm. **(E)** Primary root length of the seedlings in **(D)**. Data are shown as means ± SE (*n* = 40). Different letters indicate significant differences, as determined by one-way ANOVA (*p* < 0.05).

To assess the potential role of ABCG11 in cytokinin transport, we compared the phenotype of the *abcg11* mutant with that of the previously reported cytokinin transporter mutant *abcg14* (Ko et al., 2014; Zhang et al., 2014). In previous research, *abcg14* seedlings were grown on MGRL medium under short-day conditions (Ko et al., 2014). When the seeds derived from *abcg11/+* plants were sown and grown under the same conditions, the seedlings exhibited two different growth patterns; some seedlings had longer roots and shorter hypocotyls than others (Figure 4A). Under the same growth conditions, the *abcg14* mutant also exhibited longer roots and shorter hypocotyls than its WT background Col-4 (Figures 4A,C). Importantly, all progenies with longer roots and shorter hypocotyls from *abcg11/+* plants were homozygous for the T-DNA insertion, while the remaining seedlings either harbored the WT or *abcg11/+* genotype (Figure 4B). In addition, quantified results also revealed that the primary root length of both *abcg11* and *abcg14* mutants had significantly longer roots compared to their respective WT (*p* < 0.01, Figure 4C). These results suggested that ABCG11 and ABCG14 may function similarly in regulating root and hypocotyl growth.

We also tested how *abcg11* and *abcg14* roots responded to exogenous cytokinins when grown on half-strength MS medium. The addition of 20 nM tZ to the medium reduced the growth of primary roots of the WT Col-0 and Col-4 by about 33% but had no effect on *abcg11* or *abcg14* (Figures 4D,E), revealing that *abcg11* responds similarly as *abcg14* to exogenous cytokinin treatment. These results indicated that ABCG11 and ABCG14 have similar functions in root development.

We obtained another T-DNA allele in *ABCG11* (SALK_131624), which was previously designated *cof1-3* (Ukitsu et al., 2007) and tested its root response to tZ. Homozygous *cof1-3* plants are sterile, necessitating the use of segregating progeny, as with *abcg11*. Seedlings germinated from the seeds collected from *cof1-3/+* plants exhibited two distinct growth patterns, as observed with the progeny from *abcg11/+*: with some seedlings experiencing organ fusion (Figure 1D). Seedlings with the organ fusion phenotype were homozygous for *cof1-3* and only decrease but did not abrogate transcription of *ABCG11*, making *cof1-3* a knockdown mutant (Figure 1A). Homozygous *cof1-3* seedling roots displayed a similar response to exogenous cytokinins as *abcg11* (Figure 1E), with a modest root lengthening in both mutants relative to untreated seedlings, in contrast to the shorter roots seen in their WT upon cytokinin treatment (Figure 1F). That two independent mutant alleles of *ABCG11* exhibited a similar alteration in root growth response to cytokinin treatment, thus supporting a role for ABCG11 in cytokinin responses.

### Cytokinin signaling is enhanced in *abcg11* mutant roots upon cytokinin treatment

**Figure 5:**
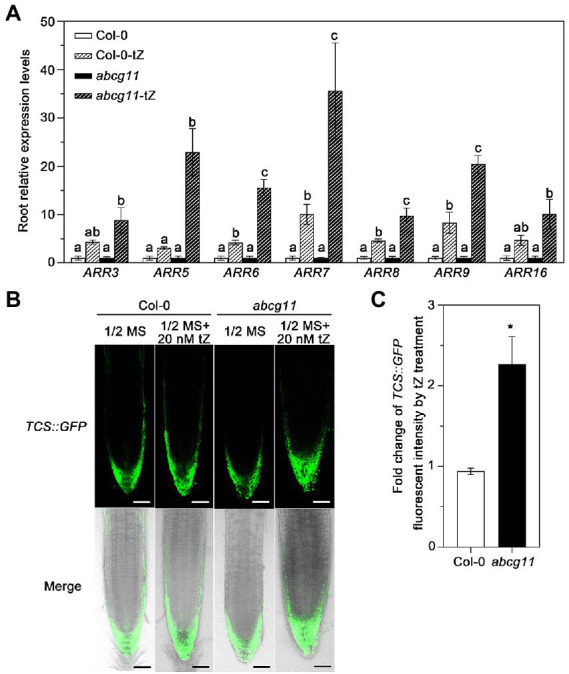
Cytokinin signaling is enhanced in *abcg11* seedlings upon tZ treatment. **(A)** Relative expression levels of *ARR*s in Col-0 and *abcg11* roots, as determined by RT-qPCR. Seedlings were grown vertically on half-strength MS medium with or without 20 nM *trans*-zeatin (tZ) plate for 12 days under long-day conditions (16 h light/8 h dark). *TUBULIN8* served as an internal control; values in Col-0 and *abcg11* under control conditions were set to 1. Data are shown as means ± SE (*n* = 4). The different letters indicate the significant difference in each gene expression levels determined by one-way ANOVA (*p* < 0.05). **(B)** Fluorescence intensity of *TCS*::*GFP* in the roots of Col-0 and *abcg11*. Scale bars, 50 mm. **(C)** Fold-change in *TCS*::*GFP* fluorescence intensity induced by tZ treatment in the root tips of WT or *abcg11* transgenic seedlings expressing *TCS*::*GFP*. Fluorescence intensity was determined using ImageJ. Data are shown as means ± SE from two independent experiments (total 10 biological replicates). Asterisks represent significant differences by Student’s *t*-test: ^*^p < 0.05.

We tested whether the levels of the cytokinin signal itself were different between the WT and *abcg11*. When treated with tZ, the root expression of type-A *ARR*s (Arabidopsis response regulators), which is induced by cytokinin (Rashotte et al., 2003; Argyros et al., 2008), was induced 9 to 20-fold in *abcg11* mutant roots, but only 3 to 10-fold in WT roots, indicating that the induction levels in *abcg11* were much higher than in the WT (Figure 5A). We also introduced the *TCS*::*GFP* reporter construct, a marker of the transcriptional output of cytokinin signaling (Zürcher et al., 2013), into *abcg11/+* plants and determined the fluorescence intensity from the green fluorescent protein (GFP) in the transgenic progeny, subjected or not to 20 nM tZ treatment. We isolated *abcg11* knockout seedlings harboring the *TCS*::*GFP* transgene based on their leaf or stem organ fusion phenotypes as above. When treated with 20 nM tZ, *TCS*::*GFP* fluorescence intensity increased more than two-fold in *abcg11* mutant roots (Figures 5B,C) but did not change significantly in WT roots, indicating that cytokinin signaling is enhanced in *abcg11* roots.

### *abcg11* and *abcg14* mutants exhibit a similar decrease in root-to-shoot transfer of cytokinin signals

**Figure 6:**
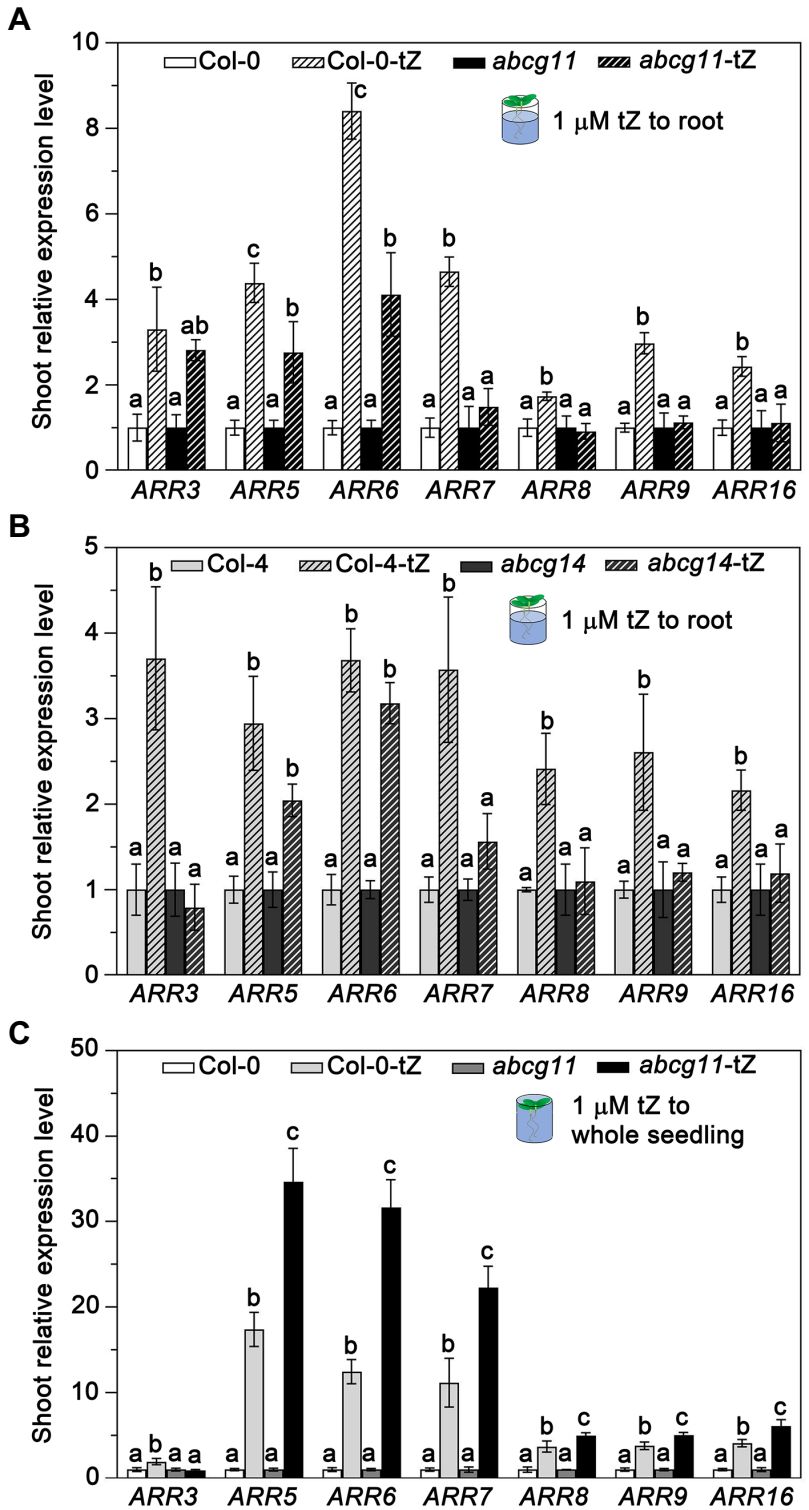
The *abcg11* and *abcg14* mutants exhibit lower root-to-shoot transfer of cytokinin signaling. **(A,B)** Relative expression levels of type-A *ARR*s in the shoots of Col-0 and *abcg11* **(A)**, and Col-4 and *abcg14* **(B)**. Only the roots of 12-d-old seedlings were soaked in half-strength MS medium or half-strength MS medium containing 1 μM *trans*-zeatin (tZ) for 1 h. The shoots were harvested for analysis of *ARR* expression levels. **(C)** Relative expression levels of *ARR*s of Col-0 and *abcg11* after tZ treatment of whole seedlings. The 12-d-old seedlings were submerged in 1 μM tZ for 30 min. *TUBULIN8* served as an internal control; values in Col-0 and *abcg11* under control conditions were set to 1. Data are shown as means ± SE (*n* = 4). Different letters in **(A)**, **(B)**, and **(C)** indicate significant differences in gene expression levels, as determined by one-way ANOVA (*p* < 0.05).

To test whether ABCG11 affects the long-distance transport of cytokinins from the root to the shoot as does ABCG14, we treated *abcg11* and *abcg14* roots with exogenous cytokinins and determined the relative transcript levels of type-A *ARR* genes in the shoot. After a 1-h treatment with 1 μM tZ, the expression levels of all *ARR* genes tested rose in WT shoots over untreated control seedlings. However, the same treatment failed to induce the expression of *ARR3*, *ARR7*, *ARR8*, *ARR9*, and *ARR16* in the shoots of the *abcg11* and *abcg14* mutants (Figures 6A,B). While *ARR5* and *ARR6* expression levels increased in the shoots of *abcg11* upon the same tZ treatment, the magnitude of the response was weaker than in WT shoots (Figure 6A).

To test whether the reduced *ARR* gene expression response in *abcg11* shoots was due to developmental problems in the shoot, we exposed entire seedlings to 1 μM tZ and measured *ARR* expression levels in shoots. After 1 h of submergence, *abcg11* shoots induced the expression of most *ARR* genes to higher levels than those of WT shoots (Figure 6C), indicating that *abcg11* shoots can perceive and respond to cytokinin signals.

To distinguish between a specific defect in cytokinin transport from the root to the shoot or a general shoot developmental defect leading to an indirect effect on phytohormone movement, we examined the root-to-shoot transfer signal of the other phytohormone, ABA. Accordingly, we incubated WT and *abcg11* roots with 100 μM ABA for 1 h and measured the relative transcript levels of ABA-induced genes in the shoots. We selected 100 μM ABA for the analysis of short-term gene expression responses as a commonly used ABA concentration (Anderson et al., 2004; Li et al., 2016). Importantly, ABA-induced genes appeared to be expressed to largely similar levels in WT and *abcg11* shoots (Supplementary Figure S1). These results suggested that the root-to-shoot transfer of cytokinins is specifically disrupted in *abcg11*, as is the case in *abcg14*.

**Figure 7:**
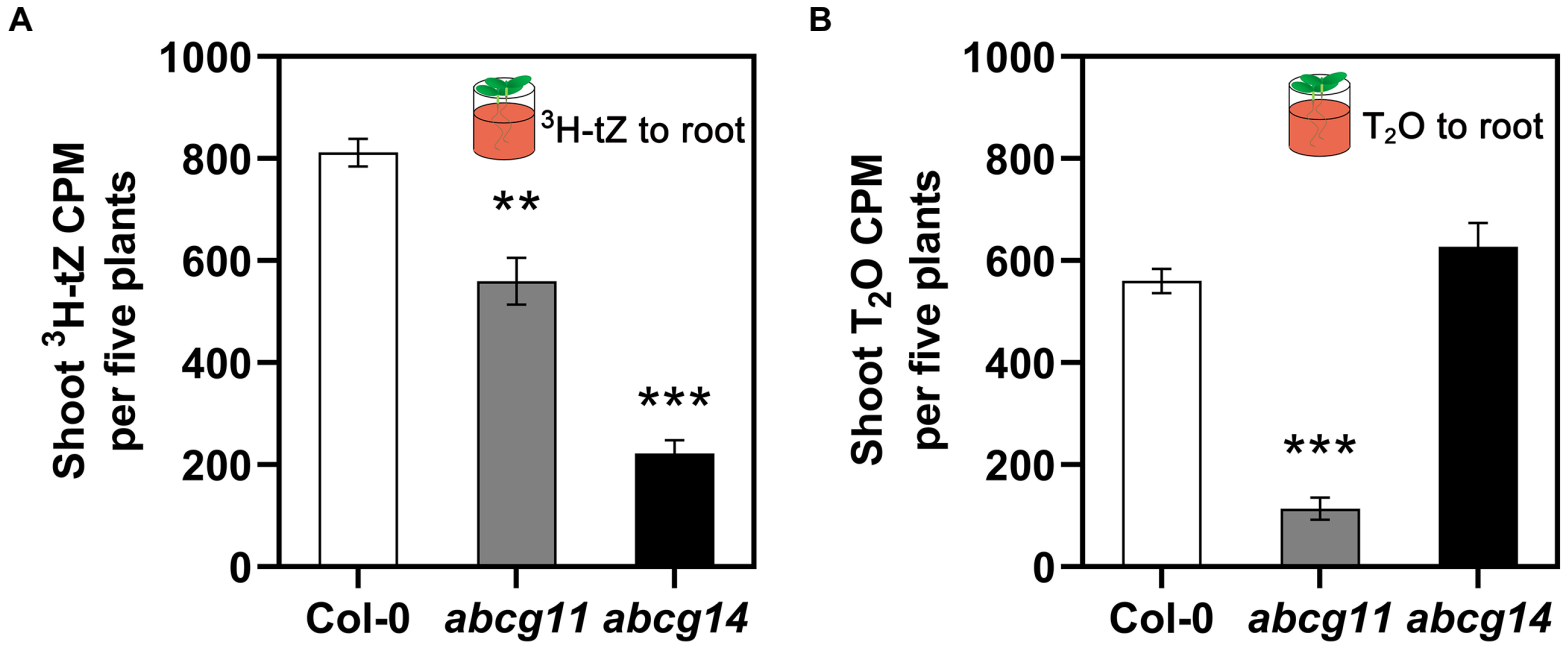
The root-to-shoot transfer of [3H]tZ and T_2_O. Only the roots of 12-day-old seedlings were treated with 50 nM ^3^H-tZ or 0.5 mCi/ml T_2_O for 1 h, and then the shoots were harvested for the assay of the transferred ^3^H-tZ or T_2_O. The error bars represent the standard deviation (SE; *n* = 6–8). The asterisks represent the significant differences between mutants and wild type by Student’s *t*-test: ^**^p < 0.01, ^***^p < 0.001.

We also directly assayed cytokinin translocation from the root to the shoot of WT (Col-0), *abcg11*, and *abcg14* plants. To this end, we specifically exposed roots to ^3^H-labeled tZ or tritiated water (T_2_O) for 1 h and measured the resulting radioactivity in the shoots. When incubated with ^3^H-tZ, we detected less radioactivity in the shoots of the *abcg11* (~69% of WT) and *abcg14* (~27% of WT) mutants than in the WT (Figure 7A). We observed no differences in T_2_O radioactivity between Col-0 and *abcg14*, while *abcg11* was characterized only 20% T_2_O radioactivity relative to the WT (Figure 7B), indicating that water transpiration is defective in *abcg11*. As we were unable to devise conditions that would bring transpiration rates in *abcg11* close to those of the WT, the possibility remains that the drop in cytokinin translocation from the root to the shoot is an indirect consequence of reduced transpiration.

### The *abcg14 abcg11* double knockout shows root phenotypes similar to those of *abcg11*

**Figure 8:**
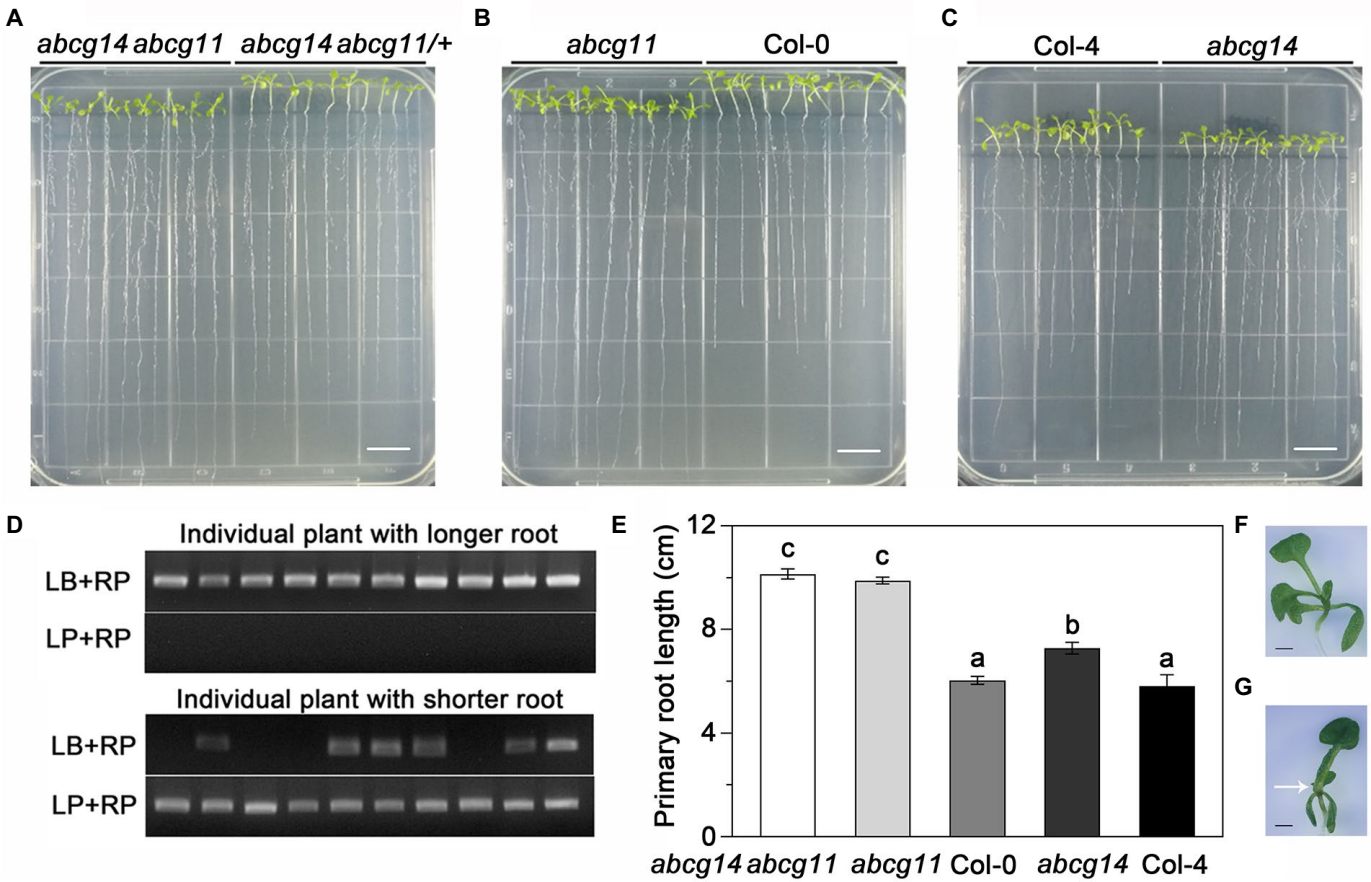
The *abcg14 abcg11* double knockout mutant exhibits a similar root length phenotype as the *abcg11* single mutant. **(A–C)** Photographs of seedlings grown vertically on MGRL medium for 12 d under short-day conditions (8 h light/16 h dark). Scale bars, 1 cm. Note that seedlings in **(A)** were rearranged according to their primary root length prior to the photograph. **(D)** Genotyping results of *abcg14 abcg11/+* and *abcg14 abcg11* seedlings shown in **(A)**. **(E)** Primary root length of the seedlings shown in **(A–C)**. Data are shown as means ± SE (*n* = 30). Different letters represent significant differences, as determined by one-way ANOVA (*p* < 0.05). **(F,G)** Photographs of *abcg14* **(F)** and *abcg14 abcg11* **(G)** seedlings. The arrow points to a site of organ fusion. Scale bars, 1.0 mm.

**Figure 9:**
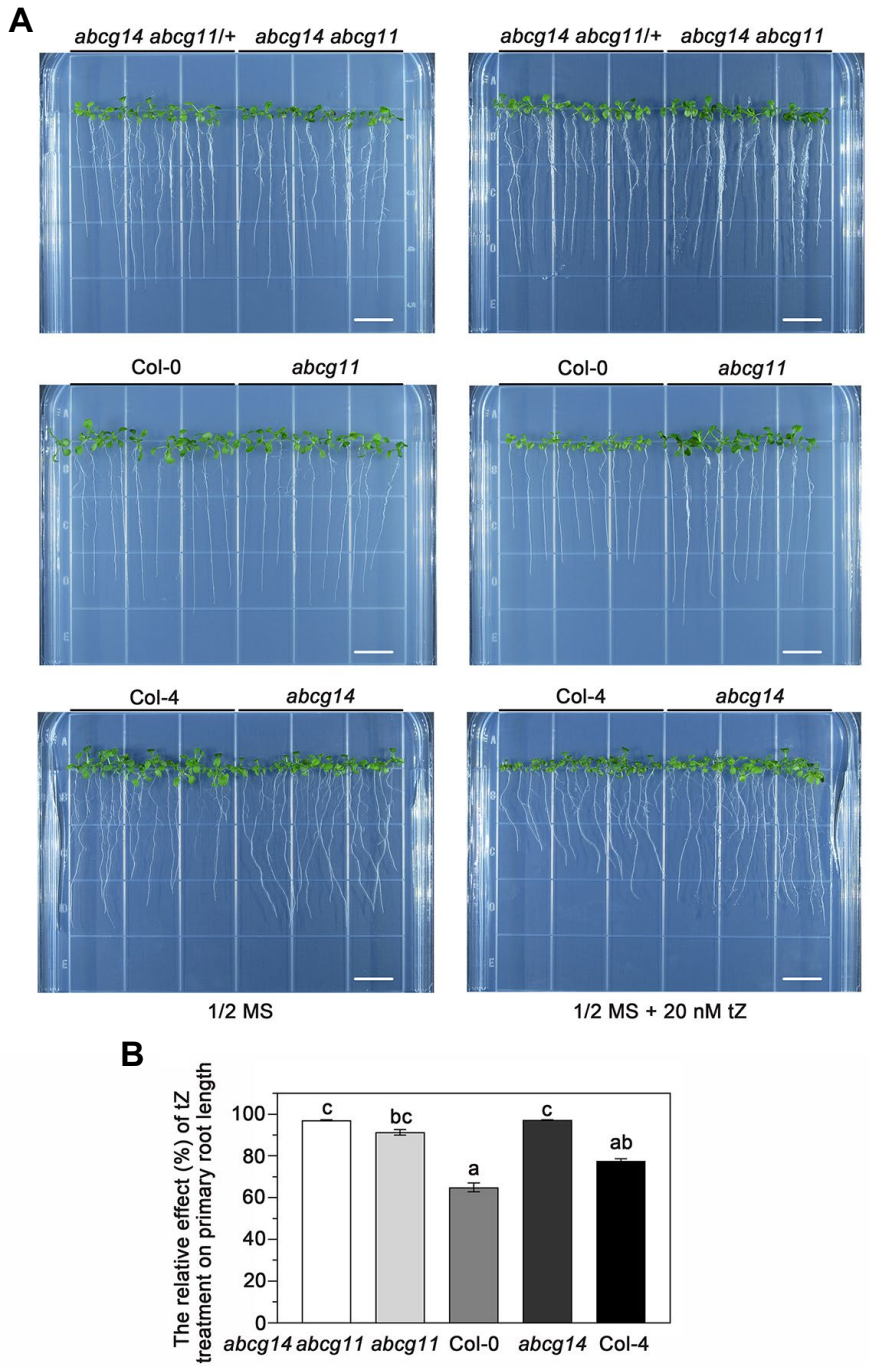
The *abcg14 abcg11* double knockout mutant is similar to *abcg11* and *abcg14* single mutants in root growth in response to cytokinins. **(A)** Seedlings grown with or without 20 nM *trans*-zeatin (tZ) treatment for 12 days under long-day conditions (16 h light/8 h dark). The photographs are representatives of four independent experiments. Scale bars, 1 cm. **(B)** Relative effect of tZ treatment on primary root growth. The relative effect was calculated as primary root length under tZ treatment normalized by that of the control treatment. Data are shown as means ± SE from four independent experiments. Different letters represent significant differences, as determined by one-way ANOVA (*p* < 0.05).

To further test the functional relationship between ABCG11 and ABCG14, we attempted to generate the *abcg14 abcg11* double mutant. As *abcg11* is sterile, we collected seeds from *abcg14 abcg11/+* plants and used them for growth analyses. We visually differentiated putative *abcg14 abcg11* seedlings from putative *abcg14 abcg11/+* seedlings based on their very different growth characteristics (Figure 8A) and confirmed the genotype of individual plants by PCR (Figure 8D), as performed earlier for the selection of *abcg11* homozygous seedlings (Figure 4B). The *abcg14 abcg11* shoots exhibited the stem and leaf organ fusion phenotypes similar to *abcg11*, and were as small as those from *abcg14* (Figures 8F,G). When grown on MGRL medium under short-day conditions, the root length of *abcg14 abcg11* homozygous seedlings was similar to that of the *abcg11* single mutant, but longer than that of *abcg14 abcg11/+* or *abcg14* single mutant seedlings (Figures 8A–E). These results suggested that *abcg14 abcg11* double knockout does not exacerbate the root developmental phenotypes of the *abcg11* single mutant. We also checked the response of *abcg14 abcg11* to exogenous cytokinins by root elongation assays. However, the primary root growth of *abcg14 abcg11* seedlings was insensitive to exogenous cytokinin treatment, similar to that observed for *abcg14* and *abcg11* (Figures 9A,B), but in contrast to their corresponding WTs (Figures 9A,B). These results indicated that the *abcg14 abcg11* double mutant does not have additional root phenotypes compared to *abcg11*.

## Discussion

ABCG11 has previously been established as an efflux transporter critical for the formation of surface coating layers such as wax and cutin (Bird et al., 2007; Luo et al., 2007; Panikashvili et al., 2007, 2010; Ukitsu et al., 2007). Since *abcg11* knockout plants exhibit very dramatic developmental defects and sterility, and because ABCG11 forms dimers with multiple partner ABCGs (McFarlane et al., 2010; Le Hir et al., 2013), we suspected that it might also participate in phytohormone transport to regulate plant development. We presented here four lines of evidence, demonstrating that ABCG11 is involved in cytokinin responses. First, the expression of many cytokinin-induced genes was different in *abcg11* compared to the WT (Figures 2A,B). Second, *abcg11* accumulated more cytokinins than the WT (Figure 3). Third, the growth of *abcg11* mutant roots was insensitive to exogenous cytokinins (Figures 4D,E, 1E,F). Fourth, the strength of the cytokinin signal was stronger in *abcg11* roots than in WT roots upon treatment with exogenous cytokinins (Figure 5). Together, these data support the idea that ABCG11 is involved in the regulation of cytokinin-mediated development.

Despite the results shown here, we did not pinpoint how ABCG11 modulates cytokinin-mediated development. ABCG11 might transport cytokinins to modulate their concentration and distribution in the root. This possibility is supported by three sets of evidences. First, *abcg11* shares many phenotypes with *abcg14* (Figure 4); ABCG14 is important for root-to-shoot cytokinin transport (Ko et al., 2014; Zhang et al., 2014) and cytokinin redistribution in shoots (Zhao et al., 2021). The similar phenotypes of *abcg11* and *abcg14* suggest that ABCG11 might collaborate with ABCG14 in transporting cytokinins. Second, the lower induction of type-A *ARR* expression in *abcg11* shoots upon root exposure to cytokinins established that the mutant has a reduced root-to-shoot translocation of cytokinin signaling, as does *abcg14* (Figure 6). Third, ABCG11 formed a heterodimer with ABCG14 (Le Hir et al., 2013). If ABCG11 is indeed involved in cytokinin transport, it exists as two distinct ABCG11-containing protein complexes (ABCG11 homodimers and ABCG11/ABCG14 heterodimers) and joins ABCG14 homodimers, which might also transport cytokinin. However, homodimers of either ABCG11 or ABCG14 alone are unlikely to be sufficient for normal cytokinin transport to the root, as the single mutants *abcg11* (with functional ABCG14) and *abcg14* (with functional ABCG11) exhibited altered responses to cytokinins in roots (Figures 4D,E) and showed altered root-to-shoot translocation of cytokinin signaling (Figure 6). The two homodimers may therefore cooperate for full normal cytokinin transport from roots to shoots, or this role is reserved for the ABCG11/ABCG14 heterodimer. In addition, it is possible that the heterodimer of ABCG11 and ABCG14 may have stronger transport activity of cytokinin, while homodimers have lower activity for cytokinin transportation. Notably, the *abcg14 abcg11* double mutant did not exhibit any additive root phenotypes relative to the *abcg11* knockout but produced longer roots than the *abcg14* single knockout (Figure 8). We suspect that this might reflect the involvement of ABCG11 in diverse functions, whereas ABCG14 is more specific. It would be interesting to further study the cytokinin transport activity of ABCG11 and ABCG14 by structure analysis and protein transport assay.

Importantly, the measured cytokinin contents did not fully support a function for ABCG11 in cytokinin transport, as cytokinin levels in both the shoot and root were higher in the *abcg11* mutant than in the WT (Figure 3). We speculate that the role of ABCG11 in the long-distance transport of cytokinin is more complex than that of ABCG14, including both direct effects as a component of cytokinin transporter and indirect effects *via* transport of its other substrates, such as the cutin/wax components. We attempted cytokinin transport assays in *abcg11* and *abcg14* seedlings and by expressing *ABCG11* and/or *ABCG14* in yeast (*Saccharomyces cerevisiae*), but did not reach a clear conclusion due to many technical problems. Further technological advances will be required to reliably track the routes of cytokinin transport in roots and the involvement of ABCG11 in this process.

An alternative possibility for ABCG11 in cytokinin transport would rely on the transport of very-long-chain fatty acids (VLCFAs). ABCG11 has been shown to be involved in transporting components of wax, cutin, and suberin, including VLCFAs (Luo et al., 2007; Panikashvili et al., 2007, 2010). Multiple VLCFA derivatives are essential components of the wax and cutin layer (Kunst and Samuels, 2003). VLCFAs were shown to alter the development of the shoot apical meristem by suppressing cytokinin biosynthesis (Nobusawa et al., 2013). VLCFAs in epidermal cells inhibit cytokinin biosynthesis in the vasculature, while a decrease in VLCFA contents raised the expression of the cytokinin biosynthesis gene *ADENYLATE ISOPENTENYLTRANS FERASE3* (*IPT3*; Nobusawa et al., 2013). If a similar mechanism exists in the root, a defect in the export of VLCFAs in *abcg11* roots may be responsible for the observed change of cytokinin signaling in *abcg11* roots. This possibility needs to be tested empirically.

Finally, we should consider the possibility that the severe developmental defects in *abcg11* might have indirectly contributed to its abnormal cytokinin responses or to affecting cytokinin concentrations *via* feedback control mechanisms. The lower water transpiration rate seen in *abcg11* (Figure 7) was in line with this possibility, but the normal transfer of ABA signaling from roots to shoots in the *abcg11* mutant (Supplementary Figure S1) disagreed with this hypothesis. Additional studies are necessary to dissect the many functions of ABCG11 and to determine the direct and indirect effects resulting from its loss of function in transport and development.

It should be noted that the root phenotypes of *abcg11* and *abcg14* mutants differ depending on the growth conditions (Figure 4). Indeed, we used a specific growth medium and photoperiod conditions to observe the phenotypes described here. Such diverse root responses indicate that plants have highly flexible mechanisms to employ cytokinins and ABC proteins to optimize root growth and adapt to their environment, which we do not fully understand. The de-coupling between cytokinin signaling and the physiological responses of *abcg11* roots also reflected the complexity of cytokinin signaling; the mutant roots were less sensitive to exogenous cytokinins (Figure 4), but cytokinin signaling was higher at the root apical meristem (Figure 5). A similar case of de-coupling was reported in plants defective in another cytokinin transporter. A knockdown in *PURINE PERMEASE14* (*PUP14*), encoding a cytokinin importer from the apoplast to the cytosol, has shorter primary roots and fewer lateral roots, which are typical phenotypes associated with cytokinin accumulation (Zürcher et al., 2016). However, *pup14* knockdown plants did not exhibit altered expression of *TCSn*::*GFP* in roots (Zürcher et al., 2016). There is therefore precedent for a defect in cytokinin transport activity that de-couples the intensity of cytokinin signaling markers, especially that of *TCSn*::*GFP*, from visible plant phenotypes. We suspect that the dual localization of cytokinin receptors to the endoplasmic reticulum and at the plasma membrane (Kubiasová et al., 2020) and the different activities between extracellular cytokinin pools and cytoplasmic cytokinin pools (Romanov and Schmülling, 2021) might contribute to the de-coupling, but this remains a speculation.

In conclusion, our results indicate that ABCG11 plays an important role in cytokinin-mediated root development in Arabidopsis, either directly as a cytokinin transporter or indirectly by transporting molecules that modulate cytokinin biosynthesis or transport. We favor the former, as ABCG11 binds with ABCG14, which is a well-known cytokinin transporter (Le Hir et al., 2013). Together with previous reports, our results suggest that ABCG11 may be involved in the transport of diverse substrates, by forming distinct dimers with diverse half-size ABCG proteins. Our findings also reveal the importance of cytokinin transport in normal root development and how much is yet to be learned about the complex mechanism of cytokinin movement and function in the root.

## Data availability statement

The original contributions presented in the study are included in the article/Supplementary material, further inquiries can be directed to the corresponding author.

## Author contributions

QY and YL conceived the research. QY and JZ conducted the experiments. MK, YT, TU, and TK conducted the cytokinin measurements and isotope transport. QY, JZ, TK, HS, and YL wrote the manuscript. All authors contributed to the article and approved the submitted version.

## Funding

This research was supported by a National Research Foundation of Korea (NRF) grant (2021R1A2B5B03001711) awarded to YL, TK, and HS were supported by Grants-in-Aid from the Ministry of Education, Culture, Sports, Science and Technology, Japan (17H06473).

## Conflict of interest

The authors declare that the research was conducted in the absence of any commercial or financial relationships that could be construed as a potential conflict of interest.

## Publisher’s note

All claims expressed in this article are solely those of the authors and do not necessarily represent those of their affiliated organizations, or those of the publisher, the editors and the reviewers. Any product that may be evaluated in this article, or claim that may be made by its manufacturer, is not guaranteed or endorsed by the publisher.

## Footnotes

^1^ https://www.takara-bio.com
