# Supplementary material for: ABCG11 modulates cytokinin responses in Arabidopsis thaliana
Source: Front Plant Sci. 2022 Jul 25;13:976267. doi: 10.3389/fpls.2022.976267 (PMC9358225; doi:10.3389/fpls.2022.976267)
Supplement: Supplementary file 1 [file Data_Sheet_1.PDF]

Table S1 Primers sequences used for RT-qPCR analysis.

Genes	Forward primer	Reverse primer
CKX3	5’-CTCAATACACAGTCAACGAGGAA-3’	5’-TCGTACATAAACCCTCTTACATGG-3’
CKX4	5’-TCGGTAACATAACCGACGAA-3’	5’-AGAATCCTCCGTTAGCGAAAC-3’
CKX5	5’-CCATGGTCCTCAAATTAGTAACG-3’	5’-TCTGAGCATCTCATCACCTCTC-3’
ARF5	5’-GGGTCAGTCGGGAGATCAAT-3’	5’-CCTTACGCATCCCACAAACT-3’
ARF6	5’-GCATCCGCAGCTTATCTGTCAGC-3’	5’-CCGCGGGAAGGTAAGGATCTTTTTG-3’
ARF8	5’-CCATGGGAGTCATTTGTGAA-3’	5’-AGTGGAAACGACTTCAAATGG-3’
ARF16	5’-TCCGTGGTGCTCAGGTATGAGG-3’	5’-CACGGGTTAACTCGCTTCACGTT-3’
ARF18	5’-TTAGCTCGGCATCAAAGCACAATG-3’	5’-GAATCGGGTACCGAGAGAAAATCCA-3’
ABI5	5’-AAACATGCATTGGCGGAGTTGG-3’	5’-CGGTTGTGCCCTTGACTTCAAAC-3’
HAI3	5’-TCCCTTTATCAACCGATCACAA-3’	5’-AGCTCCTTCGATTCGATCCA-3’
SnRK3.21	5’-AACATCTCGGGAACGATGGGTT-3’	5’-GGAGGAAGGACAGGGACGTAGTG-3’
NCED2	5’-TCTGGAATGCATGGGAATCGC-3’	5’-CGACATACACGATCCGATCAC-3’
ARR3	5’-TCTCAGCCACATCCTCGATGG-3’	5’-TCCACAAGCGAAGTTGCAGAC-3’
ARR5	5’-AGCTCAAAGATTCACACACATGC-3’	5’-TCTCCTCTCTAATGAATCCAAGTC-3’
ARR6	5’-TCCGATGCAAATTCCGTGACTG-3’	5’-AACCCACTGAATTCAATCAGCG-3’
ARR7	5’-TGAGGTCATGAGGATGGAGATTC-3’	5’-CAAGATACTGCAAAGCCCTAGTTC-3’
ARR8	5’-TCGGTCTGAAGGAGGACTAACG-3’	5’-TGCAGTCCGTTGTTGTTTGCTTC-3’
ARR9	5’-AATGGAGTCCCCACTGCAGTAG-3’	5’-TTGTTGATACTCAATGTTTGCTCC-3’
ARR16	5’-TGCAAAGTGACAACAGCAGA-3’	5’-CCAGGCATACAGTAATCGGT-3’
Tublin8	5’-GCCAGCAAAAACGCCATAATGGTG-3’	5’-GACGAAGTGGAAGTGCAGGAGGAGC-3’

Table S2 Primers sequences for genotyping.

Name	Sequence
LB	ATTTTGCCGATTTCGGAAC
LP	TTTTGGGATGTCATTTGGAAG
RP	AGTTTGAAGCAAGCGATGAT

Table S3 Primers sequences for genotyping SALK_131624 as a knock-out line or a knock-down line.

Genes	Forward primer	Reverse primer
AtABCG11	5’-ACAAGCTGGGTTTCCATGTCCTG-3’	5’-AGCTTCGGCTGTGGTAATCTTTTCC-3’
AtActin2	5’-CCAGAAGGATGCATATGTTGGTGA-3’	5’-GAGGAGCCTCGGTAAGAAGA-3’

Table S4 Cytokinin levels in roots and shoots of wild-type and *abcg11* mutant plants

Cytokinins	WT root (pmol/g FW)	*abcg11* root (pmol/g FW)	*abcg11*/WT root (%)	WT shoot (pmol/g FW)	*abcg11* shoot (pmol/g FW)	*abcg11*/WT shoot (%)
tZ	2.1 ± 0.15	**3.3 ± 0.05****	**155.6**	0.7 ± 0.04	0.7 ± 0.04	96.9
tZR	6.0 ± 0.63	8.6 ± 0.31	141.9	1.2 ± 0.17	1.0 ± 0.07	84.0
tZRPs	18.8 ± 0.74	23.2 ± 1.34	123.0	18.9 ± 0.51	19.5 ± 0.63	103.2
tZ7G	38.5 ± 0.71	**54.2 ± 5.54***	**140.9**	28.9 ± 1.01	**32.9 ± 0.69***	**114.0**
tZ9G	11.4 ± 0.52	**18.3 ±0.76****	**161.4**	6.5 ± 0.29	7.3 ± 0.14	111.6
tZOG	17.2 ± 0.58	19.4 ±1.66	112.7	11.1 ± 0.21	**9.4 ± 0.28****	**84.5**
tZROG	0.5 ± 0.05	0.7 ±0.08	145.4	0.8 ± 0.03	**1.2 ± 0.10***	**153.5**
tZ sum	94.5 ± 1.85	**127.6 ±9.11***	**135.1**	68.1 ± 0.72	72.0 ± 1.46	105.7
iP	0.357 ± 0.35	N.D.	N.D.	0.9 ± 0.07	0.9 ± 0.05	100.0
iPR	1.1 ± 0.05	1.7 ± 0.32	161.8	0.4 ± 0.08	0.4 ± 0.06	117.3
iPRPs	12.3 ± 1.3	11.7 ± 0.91	95.3	24.4 ± 4.6	25.0 ± 1.90	102.4
iP7G	80.2 ± 3.85	**120.9 ± 3.88****	**150.7**	160.8 ± 3.78	**193.6 ± 7.6***	**120.4**
iP9G	1.2 ± 0.04	**1.8 ± 0.03****	**150.0**	2.6 ± 0.11	2.8 ± 0.09	109.4
iP sum	95.1 ± 4.99	**136.1 ± 2.69****	**143.1**	189.0 ± 6.86	**222.6 ± 0.04***	**117.8**
cZ	0.9 ± 0.07	2.4 ± 1.27	268.0	0.2 ± 0.03	0.2 ± 0.01	129.4
cZR	3.2 ± 0.31	12.4 ± 8.36	381.8	0.3 ± 0.005	**0.6 ± 0.06****	**188.8**
cZRPs	12.9 ± 0.91	32.1 ± 13.85	250.1	4.1 ± 0.07	**6.0 ± 0.30****	**147.6**
cZOG	3.0 ± 0.09	3.5 ± 0.40	116.2	1.5 ± 0.18	**2.1 ±0.10***	**137.4**
cZROG	0.5 ± 0.02	**0.9 ± 0.08****	**178.8**	1.5 ± 0.02	**3.0 ± 0.06*****	**208.1**
cZ sum	20.5 ± 1.08	51.3 ± 23.00	250.2	7.6 ± 0.19	**12.0 ± 0.31*****	**158.8**

The plants were grown on the 1/2 MS plate for 12 days under long-day condition (16h/8h, day/night). Data are mean ± SE, $n = 4$. The asterisk and values in bold represent the significant difference between WT and *abcg11* by Student's *t*-test: * ($p < 0.05$), ** ($p < 0.01$), *** ($p < 0.001$). tZ, *trans*-zeatin; cZ, *cis*-zeatin; iP, isopentenyladenine; FW, fresh weight.

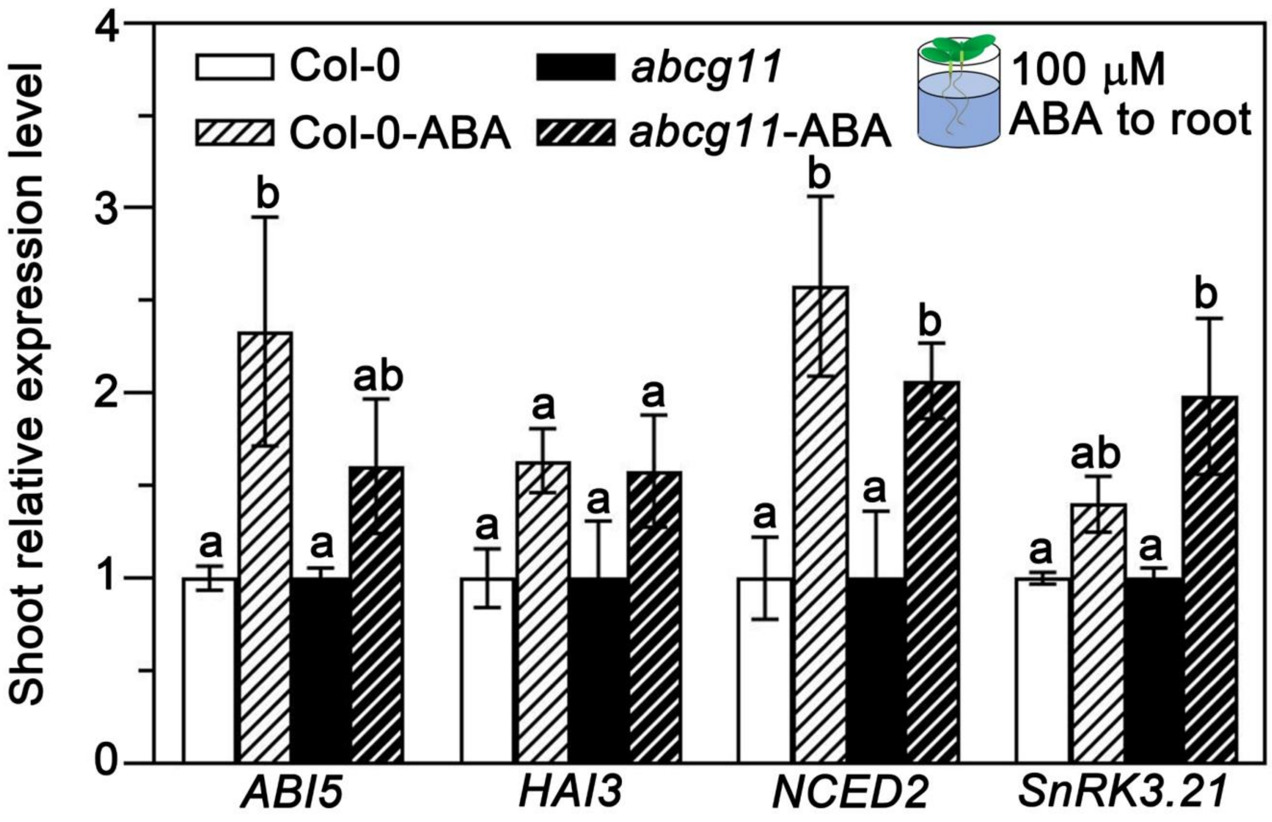

Figure S1 No difference between *abcg11* and wild type in the root-to-shoot transfer of ABA signaling.

Only the roots of 12-day-old seedlings were treated with 100 μM ABA for 1 h, and then the shoots were harvested for the assay of the expression levels of ABA induced genes. The gene expression level was normalized by housekeeping gene *Tublin8* expression level and then by the corresponding control treatment. The error bars represent the standard error (SE) (n = 4). The different letters indicate the significant differences in each gene expression levels determined by one-way ANOVA ($p < 0.05$).
